# PROMETHEUS clinical trial protocol: tailoring healthy ageing with lifestyle and nutraceuticals

**DOI:** 10.1007/s11357-026-02394-6

**Published:** 2026-07-03

**Authors:** Ajla Hodzic Kuerec, Lihuan Guan, Weilan Wang, Duarte Barros, Wejdan M. Alenezi, Jozo Grgic, Vanessa K. Wazny, Jordi Morwani-Mangnani, Mazzarine Dotou, Dingding Zhang, Tong Chen, Jiaqiu Wang, Jane Yu Ying Ong, Jian Hua Tay, Natasha To-Anan, Christian R. Sotelo, Atikah D. Binti Naseer Khan, Wester Bruggink, Sabarinath V. Nair, Saiful A. Bin Mohamed Sukor, Shivaji Rikka, Renee Chng Yee Sin, Hui Min Chong, Faiz Bin Mohamed Hashim, Sharon N. Binte Shahul Hameed, Anees B. Binte Abdul Hamid, Xin Jin, Carmina Fuster, Yong Chen, Anna Milani, Cuilin Zhang, Paul Macary, Timothy R. Singham, Karishma Sachaphibulkij, Michael Dunn, Lin Yi, Andrea B. Maier

**Affiliations:** 1 NUS Academy for Healthy Longevity, Yong Loo Lin School of Medicine, National University of Singapore (NUS), Singapore, 117456 Singapore; 2 Healthy Longevity Translational Research Programme, Yong Loo Lin School of Medicine, National University of Singapore, Singapore, 117597 Singapore; 3 Department of Human Genetics, McGill University, Montreal, QC H3A 0C7 Canada; 4 Cancer Research Program, Centre for Translational Biology, The Research Instituteof , McGill University Health Centre, Montreal, QC H4A 3J1 Canada; 5 Department of Endocrinology, Tianjin Union Medical Center, The First Affiliated Hospital of Nankai University, Tianjin, 300121 China; 6 Department of Geriatrics, The First Affiliated Hospital, Zhejiang University School of Medicine, Hangzhou, 310000 China; 7 Department of Clinical Nutrition, Hubei Cancer Hospital, Tongji Medical College, Huazhong University of Science and Technology, Wuhan, Hubei China; 8 Department of Rheumatology and Immunology, Affiliated Hospital of Zunyi Medical University, Zunyi, 563000 China; 9 Sparkd By Chi Longevity, Singapore, 068893 Singapore; 10 Global Center for Asian Women’s Health, Yong Loo Lin School of Medicine, National University of Singapore, Singapore, 119077 Singapore; 11 Department of Obstetrics and Gynaecology, Yong Loo Lin School of Medicine, National University of Singapore, Singapore, 119228 Singapore; 12 Bia-Echo Asia Centre for Reproductive Longevity & Equality (ACRLE), Yong Loo Lin School of Medicine, National University of Singapore, Singapore, 117456 Singapore; 13 Department of Microbiology and Immunology, Yong Loo Lin School of Medicine, National University of Singapore, Singapore, 117597 Singapore; 14 Life Sciences Institute, National University of Singapore, Singapore, 119077 Singapore; 15 NUS-Cambridge Immune Phenotyping Centre (NCIPC), National University of Singapore, Singapore, 117456 Singapore; 16 Department of Psychology, National University of Singapore, Singapore, Singapore; 17 Centre for Biomedical Ethics, Yong Loo Lin School of Medicine, National University of Singapore, Singapore, 117597 Singapore; 18 Abinopharm, Inc, 3 Enterprise Drive, Suite 407, Shelton, CT 06484 USA; 19 Department of Human Movement Sciences, @AgeAmsterdam, Faculty of Behavioural and Movement Sciences, Vrije Universiteit Amsterdam, Amsterdam Movement Sciences, Amsterdam, Netherlands

**Keywords:** PROMETHEUS, Healthcare, Gerotypes

## Abstract

The d emographic shift toward an older population is accelerating the prevalence of age-related diseases. Precision geromedicine represents a paradigm shift in targeting the biological processes of ageing to optimise health and healthspan, which likely requires multimodal tailored interventions. PROMETHEUS, as part of the XPRIZE healthspan semi-finalist competition, is an 8-week feasibility and exploratory study including 20 middle-aged-to-older participants who received a multimodal intervention comprising fundamental and augmented interventions. The fundamental interventions include sleep and dietary recommendations; supplementation with whey protein, creatine, and fucoidan; supervised exercise with an exergaming component (dual-task cognitive-physical training); motivational interviewing; and cognitive behavioural therapy. The augmented intervention is further personalised based on predefined gerotypes, reflecting individual ageing patterns beyond chronological age. At baseline, urolithin A, nicotinamide mononucleotide, and/or multivitamin-multimineral were prescribed to participants based on their muscle mass, VO_2_peak, and cognitive performance. At mid-intervention, interventions were adjusted based on individual response, including dose escalation and/or the addition of ergothioneine for inadequate improvement in cognition. Primary outcomes include measures of muscle strength and mass, immune function and cognitive performance, and feasibility indicators such as adherence and trial completion. Secondary outcomes include assessments of other biological, clinical, and digital biomarkers of ageing, as well as qualitative indicators of motivation and personal values. This study is aimed at investigating the recruitment feasibility, safety, adherence, and exploratory outcome trajectories of a multimodal, personalised precision geromedicine intervention protocol based on individual participant’s baseline characteristics and interim responses. These findings will also inform the design of a subsequent 12-month, randomised controlled trial evaluating personalised lifestyle and nutraceutical strategies with the potential to incorporate repurposed pharmacological agents to optimise age-related health outcomes.

## Introduction

As the global population ages, the burden of age-related diseases is creating substantial challenges for healthcare systems worldwide [1]. Central biological processes of ageing, including mitochondrial dysfunction, chronic low-grade inflammation, impaired autophagy, and immune dysregulation, among others, have been identified as modifiable targets for intervention [2]. Precision geromedicine provides a geroscience-grounded framework to optimise health and extend healthspan by targeting underlying mechanisms of ageing. Underpinning this approach, precision geromedicine aligns personalised gerointerventions with personal values, preferences, and behavioural contexts to enhance adherence and effectiveness [3]. The foundation of this approach lies in gerodiagnostics, the systematic assessment of biological, clinical, and digital biomarkers that capture an individual’s ageing trajectory and functional status [4]. These multidimensional data are synthesised into gerotypes, which classify individuals based on dominant ageing mechanisms rather than chronological age alone. Gerotypes enable mechanistically informed stratification, guide the selection of targeted gerointerventions [5], and enable continuous monitoring of treatment efficacy and dynamic adjustments based on individual responses [3]. This personalised approach model is the basis for Healthy Longevity Medicine [6, 7], termed precision geromedicine, and represents a shift from the current paradigm of reactive medicine.

Lifestyle, including diet and physical activity, can modulate fundamental biological processes of ageing [4, 6]. Regular physical activity, adherence to a healthy diet, optimised sleep, and mitigation of lifestyle risk factors (e.g. smoking) are associated with a higher lifespan and healthspan by approximately 4–10 years [7]. Nutraceuticals, encompassing a broad range of compounds, have also shown potential geroprotective effects in clinical trials in humans [8, 9]. Nutraceuticals such as whey protein and creatine can enhance muscle protein synthesis, preserve lean mass, and improve strength, with additive benefits when combined with exercise [10]. Other compounds, including fucoidan and nicotinamide mononucleotide (NMN), have been associated with immune and mitochondrial health [11, 12], ergothioneine with antioxidant and neurovascular protection [13], urolithin A with improved mitochondrial function and muscle endurance [14], and multivitamins with cognitive and metabolic health, particularly in at-risk or nutritionally deficient populations [15].

A shift from conventional unimodal therapies toward multimodal interventions is emerging, as combined strategies may exert additive effects across multiple biological mechanisms of ageing and thereby promote healthspan. Multimodal trials such as the Finnish Geriatric Intervention Study to Prevent Cognitive Impairment and Disability (FINGER) study demonstrated that combining dietary intervention, physical exercise, cognitive training, and vascular risk monitoring preserved cognitive function in at-risk older adults from the general population [16]. In addition to these findings, a population-based cohort study demonstrated that modest combined improvements in sleep, physical activity, and diet quality can achieve meaningful lifespan and healthspan benefits, suggesting that multimodal approaches compared to single domain interventions may be more effective by producing additive effects [17]. However, trials, including the Multidomain Alzheimer Preventive Trial and the DO-Health study, did not find significant improvements in clinical outcomes with their multimodal approach [18, 19]. These inconsistent findings likely reflect substantial inter-individual variability, underscoring the necessity of gerotypes with aligned corresponding gerointerventions.

Here, we present the protocol of the PROMETHEUS (PRecision gerOMedicinE: Tailoring Healthy agEing with lifestyle and nUtraceuticalS) trial, a semi-finalist in the XPRIZE Healthspan competition [20]. XPRIZE is an initiative aimed at accelerating the development of interventions that improve the function of multiple organ systems and extend healthy lifespan. This pilot single-arm clinical trial aims to explore the recruitment feasibility, safety, adherence to protocol, and exploratory efficacy of this personalised multimodal precision geromedicine intervention protocol.

## Methods

### Study design

**Fig. 1:**
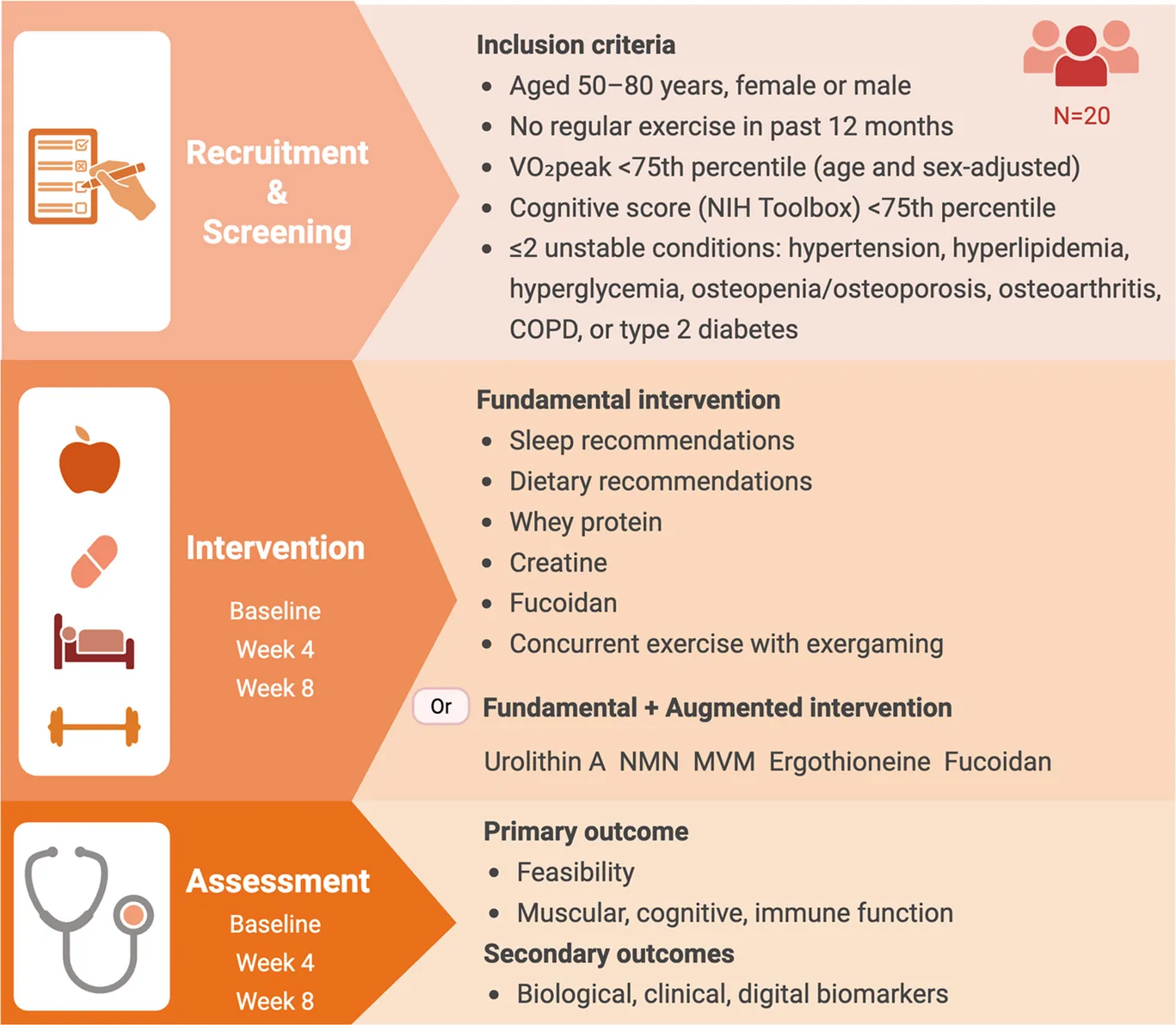
Study design. Abbreviations: COPD, chronic obstructive pulmonary disease; MVM: multivitamin-multimineral; NIH, National Institutes of Health; NMN, nicotinamide mononucleotide; VO₂peak, peak oxygen uptake. Created in BioRender

PROMETHEUS is an 8-week feasibility single-arm trial conducted at the Clinical Trial Centre of the NUS Academy for Healthy Longevity, National University of Singapore (NUS). Twenty participants received a multimodal intervention comprising fundamental and augmented interventions. Figure 1 illustrates the study design. Ethical approval for this trial was granted by the National University of Singapore Institutional Review Board (NUS-IRB-2025–393), and the study has been registered at ClinicalTrials.gov (NCT07451496).

### Participant recruitment

#### Pre-screening

Participants were recruited through different social media channels and community engagement events hosted by the NUS Academy for Healthy Longevity. Adults aged 50 to 80 years who had not engaged in regular structured exercise during the past 12 months were considered potentially eligible. Those who expressed interest were directed to the study website to complete a digital pre-screening questionnaire assessing demographics, lifestyle, and medical history after providing written informed consent for pre-screening data collection (Table 1).

**Table 1:** Criteria for recruitment

Criteria	Description
Pre-screening inclusion	Aged 50–80 years (both male and female)
	Relatively healthy (have maximum two stable chronic conditions)
	Not engaged in regular resistance or aerobic training in the past 12 months (i.e. untrained)
Inclusion	VO_2_peak below the 75th percentile for age- and sex-specific normative values
	Cognitive performance below the 75th percentile on the NIH Toolbox Cognitive Battery
	Willing and able to comply with exercise and supplementation protocols
	English-literate (can read and understand English)
	Provides written informed consent
	Deemed to have mental capacity, as assessed by the principal investigator
	Able to attend all research visits at MD11, Yong Loo Lin School of Medicine, NUS: screening (~ 2 h), 3 study visits (~ 4–5 h each, 4 weeks apart), 2 interim online visits (~ 45 min), 2 ethics interviews (~ 30 min each), and 3 supervised exercise sessions per week (45 min each)
Exclusion	Significant change in medication in the past 3 months
	History of a major cardiovascular disease (e.g. coronary artery disease, heart failure, and stroke)
	More than two stable chronic conditions (e.g. hypertension, hyperlipidaemia, hyperglycaemia, osteopenia/osteoporosis, osteoarthritis, chronic obstructive pulmonary disease, and type 2 diabetes)
	Known allergies to soy, shellfish/seaweed, mushrooms, or supplement ingredients
	Participation in another interventional clinical trial
	Current use of any study-related supplement unless willing to stop
	Any condition deemed by the principal investigator to jeopardise the safety or study compliance
	Pregnant or planning pregnancy during the study period

*NIH*, National Institutes of Health; *NUS*, National University of Singapore

#### Eligibility criteria

Participants were eligible for inclusion if they satisfied the following criteria: (1) 50 to 80 years old, (2) have not engaged in regular structured exercise during the past 12 months, (3) are relatively healthy (have maximum two stable chronic conditions), (4) have a peak oxygen uptake (VO_2_peak) below the 75th percentile for age- and sex-specific normative values [21], and (5) have cognitive performance below the 75th percentile on the NIH Toolbox Cognitive Function Battery [22].

Participants were excluded if they (1) experienced a significant change in medication within the past 3 months; (2) have a history of major cardiovascular disease such as coronary artery disease, heart failure, or stroke; (3) have more than two stable chronic conditions such as hypertension, hyperlipidaemia, hyperglycaemia, osteopenia/osteoporosis, osteoarthritis, chronic obstructive pulmonary disease, or type 2 diabetes; (4) have known allergies to soy, shellfish/seaweed, mushrooms, or any study supplement ingredients; (5) are participating in another interventional clinical trial; (6) are currently consuming any study-related supplements unless willing to discontinue them for the duration of the study; (7) have any condition that, in the opinion of the principal investigator, may compromise participant safety or study compliance; and/or (8) are pregnant or planning to become pregnant during the study period (Table 1).

#### Screening and enrolment

Participants who met the eligibility criteria and did not fulfil any exclusion criteria during pre-screening were invited to attend an in-person screening visit. At the screening visit, participants received detailed information about the study’s purpose, procedures, risks, and requirements and provided written informed consent. Recruitment commenced in July 2025 and finished in December 2025. The final participant completed the last study visit on 23 February 2026. The study is currently in the biomarker measurement and analyses phase.

Participants were assessed at baseline, during which they underwent evaluations of muscle mass and function, cognitive function, immune function, biological markers, anthropometric measurements, and clinical and digital measurements. Follow-up assessments were conducted at the mid-intervention (week 4) and at the end-of-intervention (week 8), as shown in Table 2.

**Table 2:** Overview of assessments performed at each study visit

Measurement	Pre-screening	Screening	Baseline	Week 4	Week 8
**Informed consent**	•	•			
**Lifestyle and medical questionnaire**	•				
**Cognitive function**					
National Institutes of Health Toolbox		•		•	•
**Muscle function**					
Peak oxygen uptake (VO_2_peak)		•		•	•
One-repetition maximum (1RM) test			•	•	•
**Muscle mass**					
Ultrasound			•	•	•
**Immune function**					
CD4/CD8 T cell ratio			•	•	•
Neutrophil-to-lymphocyte ratio			•	•	•
4-plex cytokine			•	•	•
Antigen-specific memory T cell response			•	•	•
Immunosenescence			•	•	•
**Biochemical blood tests**			•	•	•
**Biological analysis**					
DNA methylation profiling			•	•	•
Proteins profile			•	•	•
Metabolite profile			•	•	•
Gut microbiome			•	•	•
**Anthropometry**			•	•	•
**Body composition**					
Bioelectrical impedance analysis			•	•	•
**Cardiovascular outcomes**					
Blood pressure			•	•	•
Carotid-femoral pulse wave velocity			•	•	•
**Handgrip strength**			•	•	•
**Skin advanced glycation end products**			•	•	•
**Lifestyle monitoring**					
OURA ring			•	•	•
**Questionnaires**					
Socio-demographic survey		•			
Wellness questionnaire			•	•	•
Depression Anxiety Stress Scale-21 items			•	•	•
Three-day food diary			•		•
Quality of life					
EuroQol 5-Dimension 5-Level			•	•	•
Short Form 36 Health Survey			•	•	•
Reproductive health					
Androgen Deficiency in Aging Males			•	•	•
Brief Sexual Function Inventory			•	•	•
Female Sexual Function Index			•	•	•
Oral health					
Geriatric Oral Health Assessment Index			•	•	•
Centers for Disease Control and Prevention			•	•	•
American Academy of Periodontology			•	•	•
**Qualitative interviews**			•		•

VO_2_peak was assessed using a cardiopulmonary exercise test (CPET) conducted on an electronically braked cycle ergometer, following a standardised warm-up and an incremental ramp protocol until volitional exhaustion [21] supervised by a research assistant and/or an exercise physiologist. Prior to testing, three blood pressure readings and a temperature measurement were taken to ensure that the risk of adverse events during the test is minimal. If a participant’s blood pressure was elevated, a waiting period of 10–15 min was provided before repeating the measurement. If the blood pressure remained high, the participant was asked to reschedule the session to another day. Participants with an elevated temperature were similarly required to reschedule. Additionally, during the screening visit, cognitive function of the participants was assessed using the NIH Toolbox Cognition Battery, specifically the fluid cognition composite score and the corresponding age-corrected fluid percentage score [22].

### Interventions

All enrolled participants received a multimodal intervention comprising of fundamental and augmented interventions for 8 weeks. Fundamental interventions consisted of lifestyle interventions which were provided uniformly to all participants, whereas augmented interventions involving dietary supplements were tailored to each participant’s gerotype based on individual assessments and needs at the baseline and mid-intervention.

#### Fundamental interventions

All participants received sleep and dietary recommendations, online psychology consultation sessions with a trained clinical psychologist, and a supervised multicomponent exercise program with exergaming (dual-task cognitive-physical training). Sleep recommendations were designed to enhance both subjective sleep quality and objective sleep continuity and regularity [23]. Dietary recommendations were based on principles of the Mediterranean diet [6]. A basic informative study-tailored booklet targeting muscle mass, cognition, and immunity improvement through nutrition was provided to each participant during the initial dietary assessment interview. Dietary habits were evaluated using a nutrition assessment questionnaire conducted in person to allow a more in-depth examination, including whether participants live alone or with others, whether they usually consume home-prepared meals or dine outside, whether they do their own grocery shopping, and to capture the duration of the fasting window between the last meal of the day and the first meal on the following one. A 3-day food diary was also provided to the participants at the beginning and end of the study, with explanations on how to complete it. In addition to these interventions, a trained clinical psychologist conducted two online psychology consultation sessions with each participant to identify psychological facilitators and barriers to enhancing healthy behaviours and promoting adherence to the prescribed interventions during the interim study visits, based on behaviour change models such as COM-B [24]. These sessions also included brief cognitive-behavioural strategies and motivational interviewing.

Supervised multicomponent exercise training was conducted three times per week, with each session lasting approximately 60 min. Each session includes a warm-up, resistance training, and endurance training and concludes with dual-task cognitive–physical training. Resistance training included two sets of 8–15 repetitions across five exercises (plate-loaded seated leg extension, pin-loaded seated row, pin-loaded latissimus pulldown, barbell bench press, and dumbbell lateral raise) performed on resistance exercise machines, with a 2-min rest interval between sets. Training loads were progressively adjusted throughout the intervention to maintain the repetition range while ensuring continued overload. Endurance training was performed on a stationary bike starting at Zone 2 intensity (heart rate reserve 50–60%) and progressing to Zone 3 intensity (heart rate reserve 60–70%). Heart rate was continuously monitored using a Polar arm-band heart rate monitor (Polar Electro Oy, Kempele, Finland). At the end of the session, dual-task cognitive-physical training was performed using digital tools, including exergames and applications such as NeuroTrackerX (CogniSens Athletics Inc., Canada), SportReact reaction lights (SportReact, Croatia), SwitchedOn (SwitchedOn LLC, USA), and ClockYourself (Clock Yourself Pty Ltd., Australia). Training focused on memory and balance in the first session of each week, processing speed and motor skills in the second session of each week, and executive function and mobility in the third session of each week. Sessions were periodised and programmed to be progressively increased both in terms of physical and cognitive load.

Participants received whey protein supplementation (GOLD STANDARD 100% ISOLATE Chocolate Bliss, Optimum Nutrition, Illinois, USA) using the standard 30 g scoop as supplied by the company (each delivering ~ 25 g of protein). The dosage is adjusted according to the body weight categories as follows: 40–59 kg: 1 scoop/day, 60–89 kg: 1.5 scoops/day, and 90 kg and above: 2 scoops/day [25]. In addition, participants took micronized monohydrate creatine supplementation (1.25 g per capsule Optimum Nutrition, Illinois, USA) according to the body weight categories (40–59 kg: 4 capsules/day, 60–89 kg: 6 capsules/day, 90 kg and above: 8 capsules/day) [26] and fucoidan (2.4 g/day, SIRT6Activator®, DoNotAge.org, USA) [27].

#### Augmented intervention

**Fig. 2:**
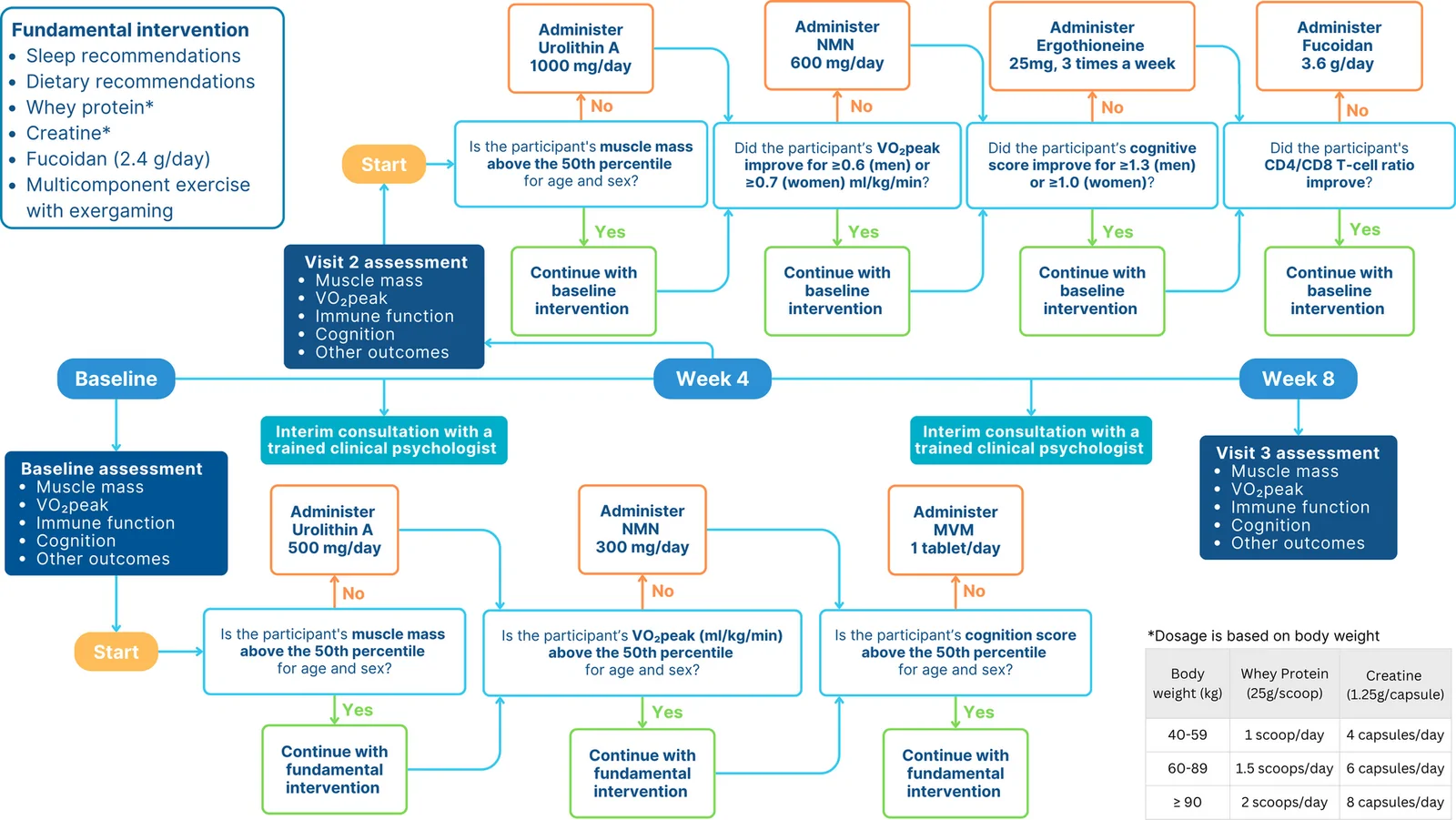
Intervention decision flowchart. Abbreviations: MVM, multivitamin-multimineral; NMN, nicotinamide mononucleotide; VO₂peak, peak oxygen uptake

This intervention was given based on participant’s assessment outcomes. At baseline, participants with (1) lower levels of muscle mass (below the 50th percentile for age and sex normative data as provided by InBody 770 analyzer (InBody Co., Ltd., Seoul, South Korea) received urolithin A (500 mg/day, Urolithin A Veggie Capsules: StanYouth® Urolithin A, Bonerge, China), (2) lower levels of VO_2_peak (below the 50th percentile for age and sex normative data) [21] received NMN (300 mg/day, AbinoNutra® NMN, Abinopharm, Inc., USA), and (3) cognitive performance below the 50th percentile for their age- and sex-group based on the NIH Toolbox Cognition Fluid Composite score [22] received a sex-specific multivitamin-multimineral supplement (1 tablet of 1.2 g/day, Centrum, USA). At the mid-intervention time point, the primary outcomes were evaluated to determine the individual progress and potentially adjust the intervention protocol. Based on the decision tree presented in Fig. 2, the intervention initiated at baseline was continued (if the improvement threshold or expected direction of changes is achieved) or adjusted (if the improvement threshold or expected direction of changes is not achieved). The augmented intervention was adjusted by (1) increasing the dose of a given supplement and/or (2) including another supplement. Specifically, if the threshold of improvements in muscle mass and VO_2_peak is not attained, the dose of urolithin A and NMN increased to 1000 mg and 600 mg, respectively [14, 28, 29]. If the threshold of changes in cognition was not achieved, ergothioneine (L-Ergothioneine Veggie Capsules: Dr.Ergo® L-ergothioneine, Shanghai EGT Synbio Group, China and Abinopharm, Inc., USA) was administrated (25 mg, 3 times per week) [30]. Lastly, if the expected direction of changes in immune function is not achieved, the dose of fucoidan was increased to 3.6 g/day [27]. The supplementation strategies and rationale are presented in Table 3.

**Table 3:** Supplementation strategies and rationale

Supplement	Baseline (visit 1)	Week 4 (visit 2)	Normative data source	Rationale
Whey protein	Dose is based on body weight as 40–59 kg: 1 scoop/day, 60–89 kg: 1.5 scoops/day, ≥ 90 kg: 2 scoops/day (25 g/scoop)	Same strategy as baseline	Not applicable	Whey protein provides additional protein supplement to improve the protein intake in Singapore (~ 0.6 g/kg from daily diet) to reach ~ 1.2 g/kg daily protein intake target [31, 32]. Protein supplement is considered safe and supports maintenance of lean muscle mass and physical function, which are key determinants of healthy ageing [33]
Creatine	Dose is based on body weight as 40–59 kg: 4 capsules/day, 60–89 kg: 6 capsules/day, ≥ 90 kg: 8 capsules/day (1.25 g/capsule)	Same strategy as baseline	Not applicable	Loading dose up to 25 g/day and maintaining dose of 4–5 g/day are considered safe [34]. It supports muscle mass [35], physical performance [36], and may also have neuroprotective effects [37]. The combination with resistance training introduced additive muscle strength benefit [38]
Fucoidan	2.4 g/day	3.6 g/day given if CD4/CD8 T cell ratio is not improved	Not applicable	Fucoidan is safe up to 4.05 g/day for 2 weeks [39]. Fucoidan, a sulphated polysaccharide derived from brown seaweed, exhibits immunomodulatory properties by enhancing innate and adaptive immune responses, including activation of NK cells and modulation of T cell function [40]. Fucoidan is associated with immune response improvement and improved natural killer cell activity [41]
Urolithin A	500 mg/day given if muscle mass is below the 50th percentile for age and sex normative data	1000 mg/day given if muscle mass is below the 50th percentile for age and sex normative data	InBody 770 analyzer internal database (InBody Co., Ltd., Seoul, South Korea)	Urolithin A is safe up to 1000 mg/day for 8 weeks [42]. As a mitophagy activator, urolithin A improves mitochondrial function and cellular health, which is critical for skeletal muscle health and cellular energy metabolism [14]. Urolithin A may improve muscle function via its antioxidative, anti-inflammatory, mitochondrial function, and cell energy-enhancing effects [43]
NMN	300 mg/day given if VO_2_peak (mL/kg/min) is below the 50th percentile for age and sex normative data	600 mg/day given if VO_2_peak (mL/kg/min) improvement is below 0.6 for men or 0.7 for women	Normative values in Chinese adults [21]. The cut-off threshold for dose escalation comes from scaled values based on the 10–20 years younger target in the planned 1-year intervention	NMN is safe up to 900 mg/day for 60 days [28]. NMN is a precursor of NAD⁺, a key cofactor in cellular metabolism [11]. Increasing NAD⁺ levels enhances mitochondrial function, energy metabolism, and physical performance, including improvements in aerobic capacity (as measured by VO₂peak) [44]
Multivitamin-multimineral	1 tablet given if cognitive performance score is below the 50th percentile for age and sex normative data based on the NIH Toolbox Cognition Fluid Composite score [45]	Continue dose as baseline	NIH Toolbox Cognition Battery database	Multivitamin-multimineral supplementation is widely used globally and helps correct subclinical micronutrient deficiencies that may impair cognitive function and immune health [45]. Adequate micronutrient status is essential for neuronal function, neurotransmitter synthesis, and immune regulation [15]
Ergothioneine	Not prescribed	25 mg (3 times per week) given if cognitive performance score improvement is below 1.3 for men or 1.0 for women [30]	NIH Toolbox Cognition Battery database	Three times 25 mg per week ergothioneine supplement was safe and induced significant improvement in previous clinical trial [30], and its combination with exercise also showed additive benefit on cognition [13]

*ATP*, adenosine triphosphate; *NAD*, nicotinamide adenine dinucleotide; *NIH*, National Institutes of Health; *NMN*, nicotinamide mononucleotide; *VO*_*2*_*peak*, peak oxygen uptake

### Primary outcomes

The primary outcomes include the feasibility of the study and longitudinal changes from baseline to mid- and end-of-intervention, assessed in muscle function (VO_2_peak and one-repetition maximum strength), muscle mass using ultrasound imaging, cognitive function using the NIH Toolbox Fluid Composite score, and immune function using the CD4/CD8 T cell ratio as indicated in the XPRIZE guideline (version 2.0) [20].

#### Feasibility assessment

The feasibility of the interventions is assessed based on six parameters:

- Participation rate, defined as the percentage of eligible participants included in the study at registration, pre-screening, and screening.
- Dropout rate, defined as the percentage of participants who withdraw during the study period, along with the reasons for withdrawal.
- Adherence to the sleep intervention, expressed as the total number and percentage of nights with ≥ 7 h of sleep time as recorded using a wearable device for continuous monitoring.
- Adherence to the supplementation intervention assessed at each study visit and calculated as the amount of supplements consumed divided by the total amount prescribed. The amount consumed is determined as the difference between the amount of supplement dispensed and the amount returned by the participants. Adherence is expressed as the total amount and percentage of supplements consumed.
- Adherence to the exercise intervention, expressed as the total number and percentage of aerobic and strength training sessions and exergaming sessions attended as recorded by the researcher.
- Adherence to dietary recommendations assessed by the 3-day food diary at baseline and end-of-intervention and the subsequential nutritional analysis.

Protocol adherence was monitored through weekly check-in to track the intervention components, including exercise attendance, supplement intake, and dual-task exercise training engagement. Overall feasibility will be assessed based on the above mentioned six feasibility parameters, which will be used as categorical variables and dichotomised based on their distribution using a cut-off point of 80%.

#### Muscle function assessments

VO_2_peak was measured using a graded exercise test conducted on a cycle ergometer. The participants first performed a standardised warm-up (5 min at 30 W). Then, the participants performed an incremental ramp protocol during which resistance was progressively increased by 1 W every 2 s until exhaustion occurred, which was defined as the point when cycling cadence fell below 60 rpm. Gas exchange and heart rate were continuously monitored throughout the test [46].

Lower limb muscle strength was evaluated using one-repetition maximum (1RM) test on the leg extension machine. Participants completed warm-up sets at 50%, 75%, and 95% of their estimated 1RM for 8–10, 3–5, and 1 repetition, respectively. Participants then performed 1RM attempts with increasing loads until they were unable to complete the lift through the full range of motion. A 3-min rest period was provided between sets.

#### Muscle mass assessment

Muscle thickness and intramuscular fat infiltration (IMAT) are assessed using a portable B-mode ultrasound system (Philips Lumify Ultrasound System) equipped with a high-frequency linear array transducer (4–12 MHz). All scans were performed by trained operators following a standardised acquisition protocol. Participants were in supine position for all measurements. For lower limb assessments, the rectus femoris and vastus lateralis were imaged at the midpoint between the anterior superior iliac spine and the superior border of patella, with the vastus lateralis assessed on the lateral aspect of the thigh. At least two high-quality images were acquired at each muscle site and subsequently analysed using the MuscleSound software (Denver, CO, USA).

#### Cognitive function assessment

Cognitive performance was evaluated using the NIH Toolbox Cognition Battery (fluid cognition composite score and age-corrected percentage score in the population) [47] administered via a tablet, and conducted in a quiet room with no distractions, and only one researcher was allowed to carry out the assessment. The composite measure captures subdomains including working memory, attention, executive function, episodic memory, and processing speed [22].

#### Immune function assessment

Immediately after venipuncture, 250 μL of fasting fresh whole blood was aliquoted from the K2-ethylenediaminetetraacetic acid (K2-EDTA) tube (Greiner Bio-One, Kremsmünster, Austria) for assessment of immunosenescence by quantifying the CD4/CD8 T cell ratio using BD TruCount™ tubes in conjunction with a BD CD4/CD8 reagent cocktail (Becton Dickinson, Franklin Lakes, NJ, USA), in accordance with the manufacturer’s instructions. Data acquisition was performed on a BD LSRFortessa™ Symphony A5 flow cytometer (BD Biosciences, San Jose, CA, USA), and data were analysed using FlowJo™ software (FlowJo, LLC, Ashland, OR, USA).

### Secondary outcomes

The secondary outcomes include the longitudinal changes from baseline to mid- and end-of-intervention in the biological, clinical, and digital biomarkers of ageing covering physical and mental health.

#### Biological sample collection and analysis

From overnight fasting peripheral blood drawn at each study visit, five fractions were collected: whole blood, serum, plasma, peripheral blood mononuclear cells (PBMCs), and buffy coat. Blood samples collected in K2-EDTA tubes and serum separator tubes (SST™) (BD Vacutainer®, Becton Dickinson) were centrifuged at 1200 × g for 15 min with soft brake to obtain plasma and serum, respectively. Prior to centrifugation, whole blood was aliquoted from the K2-EDTA tube and will be used for the measurement of nicotinamide adenine dinucleotide (NAD) concentrations using the Q-NADMED Blood NAD + and NADH Assay Kit (NADMED Ltd., Helsinki, Finland), according to the manufacturer’s instructions. Plasma will be used for proteomics profiling using SomaScan® 11 K Assay (SomaLogic Operating Co., Boulder, CO, USA). Untargeted metabolomics in plasma will be performed using a Vanquish UHPLC system coupled to an Orbitrap Exploris™ 120 high-resolution mass spectrometer (Thermo Fisher Scientific, Germany), with chromatographic separation on a Hypersil Gold column (100 × 2.1 mm, 1.9 μm; Thermo Fisher Scientific, USA) and an ACQUITY UPLC BEH Amide column (Waters, Ireland). Serum was collected for quantification of cytokines interleukin-6 (IL-6), IL-10, tumour necrosis factor-alpha (TNF-α), and IL-1β using the Cytokine 4-Plex A assay (Quanterix, Billerica, MA, USA) according to the manufacturer’s instructions, with detection performed by multiplex immunoassays on the Luminex® xMAP® platform (Luminex Corporation, Austin, TX, USA). Biochemical blood tests, comprising haematology, liver and renal function profiles, lipid profile, glucose, glycated haemoglobin (HbA1c), insulin, and high-sensitivity C-reactive protein (hs-CRP), were conducted by an accredited clinical laboratory. The neutrophil-to-lymphocyte ratio (NLR) was derived from biomedical blood tests to assess systemic inflammatory status. The PBMCs were isolated from whole blood collected in acid citrate dextrose solution A (ACD-A) tubes (BD Vacutainer®, Becton Dickinson) through density-gradient centrifugation using Ficoll-Paque™ PLUS (Cytiva, Marlborough, MA, USA), cryopreserved in foetal bovine serum (FBS) (Gibco, Thermo Fisher Scientific, Waltham, MA, USA) containing 10% dimethyl sulfoxide (DMSO) (Sigma-Aldrich, Merck KGaA, Darmstadt, Germany), and stored in liquid nitrogen for downstream immune-function assays.

Cryopreserved PBMCs were thawed and rested for 2–4 h at 37 °C, 5% CO₂ in complete RP-10 medium comprising RPMI-1640 supplemented with 10 mM L-glutamine, 10% FBS, 100 U/mL penicillin, 100 µg/mL streptomycin, and 10 mM HEPES (all Gibco). Cells were seeded at 2 × 10^5^ cells/well in 96-well U-bottom plates (Corning, NY, USA) and stimulated with 1 µg/mL CEF peptide pool (cytomegalovirus, Epstein-Barr virus, and influenza virus) to assess antigen-specific memory T cell responses. Vehicle control (< 0.1% DMSO) and positive control using Dynabeads® Human T-Activator CD3/CD28 (Gibco) at a 1:1 bead-to-cell ratio were included. After 24 h of incubation, supernatants were collected following centrifugation at 400 × g for 5 min. Interferon-gamma (IFN-γ) levels were quantified in the supernatants using a Human IFN-γ ELISA kit (Invitrogen, Thermo Fisher Scientific, Waltham, MA, USA) and read at 450 nm on an Infinite 200 PRO M Plex plate reader (Tecan, Switzerland). Immunophenotyping will be performed in the cryopreserved PBMCs to evaluate immunosenescence status.

Buffy coat fractions were isolated from the whole blood for genomic DNA extraction using QIAamp® DNA Blood Mini Kit (Qiagen, Germany) following the manufacturer’s instructions. Genomic DNA purity was assessed using NanoDrop™ spectrophotometer (Thermo Fisher Scientific, USA), with acceptable samples defined by an A260/A280 absorbance ratio between 1.6 and 2.1. DNA concentration was measured using Qubit™ 4.0 Fluorometer (Thermo Fisher Scientific, USA). DNA methylation profiling was conducted using the Infinium® MethylationEPIC v2.0 BeadChip (Illumina, San Diego, CA, USA) following the Infinium® HD Assay protocol. Briefly, 500 ng of the genomic DNA underwent bisulfite conversion, during which unmethylated cytosines were deaminated to uracils, while methylated cytosines remain unchanged. The converted DNA was then amplified, enzymatically fragmented, precipitated, and resuspended in hybridization buffer prior to hybridization onto the BeadChip, where locus-specific probes interrogated over 935,000 CpG sites at single-base resolution. After hybridization, non-specific DNA was removed through washing, followed by single-base extension with fluorescent labelling. BeadChips was then scanned using the Illumina iScan System (Illumina, San Diego, CA, USA), and DNA methylation levels were quantitatively determined based on the resulting fluorescence signals. DNA methylation age will be derived, including PCHorvathAge, PCHannumAge, PCGrimAge, PCPhenoAge [48], GrimAge2 and GrimAge2 surrogates [49], DunedinPACE [50], Ying clocks [51], and SystemsAge [52].

Stool samples were collected using the DNA/RNA Shield™ Fecal Collection Kit (Zymo Research, USA), which preserves and stabilizes microbial nucleic acids at ambient temperature during collection and transport. Participants received a stool collection kit along with written instructions, where they were asked to return the samples within 24 h of collection. Samples were stored in a − 20 °C freezer upon receiving. Short-read shotgun metagenomics sequencing will be performed to characterize gut microbiome composition and functional capacity. Genomic DNA will be extracted from each sample using a Maxwell® HT Fecal Microbiome DNA Kit (Promega Corporation, Madison, WI, USA) according to the manufacturer’s instructions. Extracted genomic DNA will be quantified using a fluorometric method with the Qubit™ dsDNA HS Assay Kit (Thermo Fisher Scientific, Waltham, MA, USA), and its integrity will be assessed using 1% agarose gel electrophoresis. For shotgun metagenomic library preparation, 1 μg of genomic DNA will be randomly fragmented to an average size of approximately 350 base pairs (bp) using a high-throughput focused-ultrasonicator (Covaris, Woburn, MA, USA). Sequencing libraries will be prepared using the Universal DNA Library Prep Kit for Illumina® V3 (Vazyme Biotech, Nanjing, China), following standard procedures including end repair, A-tailing, sequencing adaptor ligation, purification, and polymerase chain reaction (PCR) amplification. Libraries passing quality control will be carried forward for sequencing. Following library construction, fragment size distribution and library integrity will be evaluated using Advanced Analytical Technologies, Inc. (AATI) fragment analysis (Agilent Technologies, USA). Libraries with the expected insert size will be quantified by quantitative PCR. Qualified libraries will be pooled based on effective concentration and target sequencing depth, and sequencing will be performed on the Illumina NovaSeq X Plus platform (Illumina, San Diego, CA, USA) using paired-end 150 bp (PE150) chemistry. Taxonomic and functional annotation will be performed using DIAMOND software [53].

All excess plasma, serum, buffy coat, PBMCs, and stool samples were biobanked for future research subjected to IRB approval and participant consent.

#### Anthropometric measurements

Waist and hip circumferences were measured using SECA Bodymeter 206 (SECA GmbH & Co. KG, Hamburg, Germany). For waist circumference measurement, the measuring tape was positioned two finger widths above the umbilicus. Hip circumference measurement was recorded at the midpoint or widest region of the buttock.

#### Body composition analysis

Height (cm) and weight (kg) were measured using InBody Stadiometer BSM370 (InBody Co., Ltd., Seoul, South Korea). Body composition, including fat and lean mass, was evaluated via a bioelectrical impedance analysis using the InBody 770 analyzer (InBody Co., Ltd., Seoul, South Korea). Participants were instructed to stand barefoot on the device platform and follow the on-screen prompts to ensure standardised measurements conditions [54].

#### Cardiovascular assessment

Blood pressure and arterial stiffness were measured after 15 min of supine rest using SphygmoCor-XCEL device (AtCor Medical Pty Ltd., Sydney, Australia). Brachial systolic and diastolic pressures were measured using an automated cuff. Carotid-femoral pulse wave velocity (cf-PWV) was measured by applying a tonometry probe to the carotid artery and inflating a thigh cuff. Each measurement was performed up to 10 attempts to capture regular pulses.

#### Handgrip strength test

Handgrip strength was assessed using a Jamar Plus + dynamometer (Sammons Preston Rolyon, USA). Participants were requested to stand upright with the elbow flexed at 90° and the forearm in a neutral position. Maximum grip force was recorded in three attempts per hand, with a 60-s rest interval between alternate attempts [55].

#### Skin advanced glycation end product assessment

Skin accumulation of advanced glycation end products (AGEs) was assessed non-invasively using AGE Reader device (DiagnOptics Technologies B.V., Groningen, Netherlands). Measurements were performed on the dominant forearm that was cleaned with an alcohol wipe before placing it on the device. Three consecutive readings were taken per participant [56].

#### Lifestyle monitoring

Biometrics data [57] were collected with an OURA ring (Oura Health Ltd., Oulu, Finland), which was provided to participants at baseline and worn throughout the study. This device passively monitors physical activity (daily steps and activity intensity) and sleep duration throughout the intervention period. Data were synchronized via the OURA app and used to monitor adherence to sleep and physical activity recommendations, as well as to track behavioural changes over time.

#### Questionnaires

Sociodemographic characteristics were obtained using self-reported questionnaires. Wellness status was assessed using a wellness questionnaire. Mental wellbeing including depression, anxiety, and/or stress levels was assessed with Depression Anxiety and Stress Scale (DASS-21) [58]. Food intake was assessed using a 3-day food diary completed by participants after the baseline and end-of-intervention visits. Participants recorded all foods and beverages consumed on two weekdays and one weekend day [59]. Quality of life was assessed with the 36-Item Short Form Survey (SF-36) [60] and EuroQol-D5-5L (EQ-5D) [61]. Reproductive health was assessed by Androgen Deficiency in Aging Males (ADAM) [62] and Brief Sexual Function Inventory (BSFI) [63] questionnaire for males and Female Sexual Function Index (FSFI) [64] questionnaire for females. Oral health status and mucosal screening was conducted using the Geriatric Oral Health Assessment Index (GOHAI) [65] and the Self-reported Oral Health Questionnaire developed by Centers for Disease Control and Prevention (CDC) and the American Academy of Periodontology (AAP).

#### Qualitative assessment

Semi-structured qualitative interviews were used to explore both participants’ experiences, motivation, and views of trial participation and their engagement with (and evaluation of) precision geromedicine programmes more generally. These data assist in identifying how the trial design and the clinical translation of interventions can be personalised to participants’ experiences, values, and concerns, supporting ethical practice and conduct of research. Interviews lasting between 30 and 60 min were conducted at two time-points: at the point of recruitment and at the completion of the study. Reflexive thematic analysis on transcripts was undertaken to identify key themes in ways that ensure interpretive depth, transparency about analytical decisions, and researcher reflexivity [66, 67]. The interview topic guide was shaped by insights into motivation and awareness from interview studies conducted locally [68, 69] but extends these to also explore personal values and concerns.

#### Safety assessments

Information regarding the occurrence of adverse events (AEs), either reported by the participants or inquired by researchers, were systematically captured in digital database throughout the study. Given the concurrent administration of multiple supplements, safety monitoring was implemented throughout the study. All selected supplements have established safety profiles at the doses used (Table 3). Participants with any medical conditions deemed by the principal investigator (PI) as potential contraindications were excluded at screening based on medical history review. Any adverse events were recorded with temporal relation to the intervention period. Should clinically significant intolerability occur, dose adjustment, temporary discontinuation, or permanent cessation of the respective supplement was at the PI’s discretion. All collected AEs were recorded according to the Medical Dictionary for Regulatory Activities (MedDRA) and graded according to CTCAE (version 6.0) [70]. Duration (start and stop dates and times), seriousness, severity/grade, outcome, concomitant medication use, action taken, and relation to study intervention were recorded. An AE was considered a serious adverse event (SAE) if it fulfilled the International Council for Harmonization (ICH) Good Clinical Practice (GCP) guideline criteria based on the PI’s clinical judgement. Each SAE is required to be reported within 24 h of learning of its occurrence. The PI is required to complete the SAE Report Form, assess the causal relationship to study intervention, and send the completed form to the National University of Singapore Institutional Review Board and to Singapore’s Health Sciences Authority [71].

#### Sample size

This study was designed to assess the preliminary effectiveness and feasibility of a precision geromedicine multimodal intervention. For the semi-finals stage, the XPRIZE guidelines focused on developing a small pilot study including around 5 to 20 participants. We opted for the larger sample size and, therefore, 20 participants were included. This sample size reflected the feasibility considerations and provided preliminary data on recruitment rates and outcome variability within the target population [20].

### Statistical and analytical plans

Adherence to each intervention component (sleep, dietary recommendation, supplementation, and exercise) will be analysed descriptively. Adherence will be summarised across the intervention period using mean and standard deviation (SD) or median and interquartile range (IQR) where appropriate. Adherence will be additionally categorised using a predefined threshold of ≥ 80% to indicate adequate adherence. The proportion of participants meeting this threshold will be reported for each intervention component.

Changes in outcome measurements at the mid-intervention and the end-of-intervention compared to baseline will be calculated for each participant. Continuous outcomes will be summarised as mean changes with 95% confidence intervals (CIs) for approximately normally distributed data or as median changes with interquartile ranges (IQRs) for non-normally distributed data. Within-participant changes from baseline to each follow-up timepoint will be assessed using paired t-tests or Wilcoxon signed-rank tests, as appropriate.

To evaluate longitudinal changes across all study timepoints while accounting for within-participant correlation, linear mixed-effects models will be applied for continuous outcomes. Timepoint will be included as a fixed effect, and participant ID will be included as a random effect. Estimated marginal means and corresponding 95% CIs will be reported for each timepoint, together with model-based contrasts comparing mid- and end-of-intervention with baseline.

To account for multiple testing across outcome measures, the Benjamini–Hochberg procedure will be applied to control the false discovery rate. All statistical tests will be two-sided, given the small sample size (*n* = 20), *p* values will be interpreted descriptively and hypothesis-generating only, and 95% CIs will be reported where applicable to support estimation of effect magnitude and precision.

Data will be visualized in spaghetti plot to display the changes during the intervention trajectory at individual level and the group level. All data analysis and visualization will be performed using R.

## Discussion

PROMETHEUS is the first feasibility study to assess the implementation of a multimodal precision geromedicine intervention based on gerotypes with dynamic personalised adjustments. By combining structured lifestyle modification, targeted nutraceuticals supplementation, and adaptive dose/intensity adjustments based on mid-intervention responses, this study tests the hypothesis that individualised, mechanism-informed programmes can more effectively target the biological processes of ageing than single-modality or ‘one-size-fits-all’ approaches. Focusing on relatively healthy, middle-aged to older adults allows the evaluation of whether such an approach can be deployed before the onset of major age-related diseases and significant functional decline, within a window when modifying ageing trajectories may yield substantial long-term healthspan gains. The inclusion of multisystem primary outcomes such as immune function, cognitive performance, and muscle strength, together with qualitative insights and a broad panel of biological, clinical, and digital biomarkers of ageing, provides an integrated assessment of the intervention’s effects across key hallmarks of ageing.

The limitations of this trial are the small sample size, the relatively short intervention duration, and the absence of a control arm. Given the short intervention period and single-arm nature, observed changes in biomarkers or functional assessments will be interpreted as exploratory evidence of short-term biological responses. In addition, participants were relatively healthy, English-literate volunteers able to commit to frequent visits and supervised training, which may limit generalisability to more diverse or socioeconomically constrained populations. The complexity of the multimodal protocol may also pose challenges to scalability outside specialised centres, particularly in settings with limited access to equipment, advanced laboratory platforms, or trained personnel.

Despite these limitations, the results of this trial provide critical proof-of-concept evidence regarding the acceptability, adherence, safety, and preliminary efficacy of a multimodal precision geromedicine model. Demonstrating that such an intervention is feasible, safe, and capable of producing meaningful improvements in muscle, cognitive and immune function alongside favourable shifts in biological and digital ageing markers would support the development of larger, randomised controlled trials. Future work should test streamlined, scalable versions of this model in more heterogeneous populations, explore cost-effectiveness, and implementation strategies in routine clinical practice.

## Data Availability

Data generation and analysis are ongoing. For further inquiries, please contact the corresponding author.
